# Influence of Disordered Functions of the Skin

**Published:** 1849

**Authors:** John Jackson


### ARTICLE V.

*Influence of Disordered Functions of the Skin in producing Fevers.* Extracted from a paper read before the Union Medical Society of Northern Indiana. By John Jackson, M.D.

Investigations with regard to the remote and proximate causes of fever have, at all times, engaged the attention of the medical profession. Malaria, according to the prevailing opinion, is a specific agent, capable of producing the class called bilious, intermittent, and remittent fevers. But have we not otl er agents to which to ascribe the production of fever without assigning unknown and doubtful causes? Are not the different electrical conditions of the atmosphere, moisture, cold, etc., agents active enough to disturb the equilibrium of the circulation and with it that harmonious action of the nerves so necessary to perfect health? Have we not, during the winter, a season unfavorable to the production of malaria, fevers presenting precisely the same phenomena as those called intermittent and bilious? and are they not treated successfully by the same kind of medicine.

We know that during winter as well as summer, if the functions of the skin are disturbed so as to suppress perspiration, large quantities of carbon are retained in the blood, as is evident by its being found in excess, combined with other substances in the excretions.

Superabundance of carbon in the system, as is well known to medical men, is deleterious in its effects.

This redundance of carbon in the blood may, at any time or season, be occasioned by the quick closing of the pores of the skin—especially is this the case during warm weather; for, during the summer and fall, the skin is most active, and the lungs less so, for the reason that the quantity of oxygen in the inspired air is less in warm than in cold weather. In summer, therefore, the carbon cannot be carried off by means of the lungs in anything like the same proportion as during winter.

Again, all writers upon the subject of fever say it is the effect of irritation, seated, as most of them believe, in the capillaries. Allowing it to be in the capillaries, we would ask why may not fever—especially intermittent and bilious—be produced simply by an excess of carbon in the blood keeping up a coastant irritation in the capillary nerves.

Upon the first onset of most disease, patients exhibit all the symptoms of fever; that is, they present an irritability of pulse, congested vessels, and preternatural heat. These are the constant symptoms of fever; but if the action of the skin is restored, all the alarming symptoms quickly disappear, and with them the fever.

Fevers are always ushered in by a sense of nervous depression. A chill never occurs until the blood recedes from the surface. We consider, therefore, that the disturbed functions of the skin, produced by cold may be a frequent cause of fever, and that the proper treatment is to arouse its healthy action and allay nervous irritability. In the early stages this can be done by the application of heat alone; its constant effects being to stimulate the skin to action. Moreover, every practitioner knows that many of the fevers called bilious are cured by rest alone; or in other words, the patient gets well when the cause—a superabundance of carbon—is carried off by the action of the skin, liver, and other organs, or burned up in the body, as healthy action is restored.

In a word, what is an increased action of the capillaries to rid the system of this noxious matter, which first caused the irritation and which we conceive to be principally carbon.

We are not writing particularly upon the subject of fever, we will therefore say no more upon its cause or pathology, farther than to state that we have no faith in the common doctrine of a supposed agent called malaria producing them. We hope that writers upon the subject may hereafter be more philosophical; for so long as men follow phantoms of their own or other men's creation, they will never arrive at the true cause of this or any other disease. It may be asked if malaria is not a most active agent in producing that form of fever called bilious, why is it that we have so few cases of that character in old settled districts? The causes are many—first, the people are generally more regular in their habits. They have better dwellings, are better clothed, better fed, have plenty of fruits abounding in acids, the base of which is oxygen. They generally have an abundance of culinary vegetables all of which contain potash in some form.

In old settled countries they have none of that superincumbent vegetable matter which covers the surface in new countries, preventing the sun's rays from acting upon the soil, and favoring the consumption of oxygen and the formation of carbonic acid gas.

---

